# Discovering lncRNA mediated sponge interactions in breast cancer molecular subtypes

**DOI:** 10.1186/s12864-018-5006-1

**Published:** 2018-09-04

**Authors:** Gulden Olgun, Ozgur Sahin, Oznur Tastan

**Affiliations:** 1 Department of Computer Engineering, Bilkent University, Ankara, 06800 Turkey; 2 Department of Molecular Biology and Genetics, Faculty of Science, Bilkent University, Ankara, 06800 Turkey; 3 Faculty of Engineering and Natural Sciences, Sabanci University, Tuzla, Istanbul, 34956 Turkey

**Keywords:** lncRNA mediated sponges, ceRNA interactions, noncoding RNA, miRNA, lncRNA, Partial correlation analysis, Kernel conditional independence test

## Abstract

**Background:**

Long non-coding RNAs (lncRNAs) can indirectly regulate mRNAs expression levels by sequestering microRNAs (miRNAs), and act as competing endogenous RNAs (ceRNAs) or as sponges. Previous studies identified lncRNA-mediated sponge interactions in various cancers including the breast cancer. However, breast cancer subtypes are quite distinct in terms of their molecular profiles; therefore, ceRNAs are expected to be subtype-specific as well.

**Results:**

To find lncRNA-mediated ceRNA interactions in breast cancer subtypes, we develop an integrative approach. We conduct partial correlation analysis and kernel independence tests on patient gene expression profiles and further refine the candidate interactions with miRNA target information. We find that although there are sponges common to multiple subtypes, there are also distinct subtype-specific interactions. Functional enrichment of mRNAs that participate in these interactions highlights distinct biological processes for different subtypes. Interestingly, some of the ceRNAs also reside in close proximity in the genome; for example, those involving HOX genes, HOTAIR, miR-196a-1 and miR-196a-2. We also discover subtype-specific sponge interactions with high prognostic potential. We found that patients differ significantly in their survival distributions if they are group based on the expression patterns of specific ceRNA interactions. However, it is not the case if the expression of individual RNAs participating in ceRNA is used.

**Conclusion:**

These results can help shed light on subtype-specific mechanisms of breast cancer, and the methodology developed herein can help uncover sponges in other diseases.

**Electronic supplementary material:**

The online version of this article (10.1186/s12864-018-5006-1) contains supplementary material, which is available to authorized users.

## Background

Advances in sequencing technologies have revealed that there is a large number of RNAs that do not encode proteins [1]. One class of non-coding RNAs (ncRNAs) comprises microRNAs (miRNAs) that repress gene expression by preferentially binding the complementary sequence of their target mRNAs [2]. miRNAs play crucial roles in regulating gene expression programs in the normal cell, and their aberrant expression contributes to pathogenesis in several diseases, including cancer. To date, a large number of miRNAs have been shown to be associated with cancer progression, drug resistance or metastasis [3–7].

Another major class of non-coding RNAs is long non-coding RNAs (lncRNAs) that are longer than 200 nucleotides. Although the function of the vast majority of lncRNAs remains to be identified, accumulating evidence suggests that they are highly involved in regulating cellular and pathological processes [8, 9]. Deregulations of several lncRNAs have also been associated with cancer [10, 11].

Recent work has provided evidence for an emerging regulatory role of lncRNAs. According to the ceRNA hypothesis [12], lncRNAs can act as ceRNAs. By sequestering miRNAs, lncRNAs can reduce the number of miRNAs available for the target mRNA [12]; in this way, they indirectly prevent the target gene repression, acting like a sponge [13–15]. lncRNA-mediated sponge interactions and their protein-coding targets have been investigated in gastric cancer [16], glioblastoma multiforme [17], pancreatic cancer [18], ovarian cancer [19] and in breast cancer [20].

Identifying sponges by experimental means is a challenge, and the experimental datasets are not available in different contexts such as cancer. Few studies attempt to identify lncRNA sponges computationally. SpongeScan uses a sequence-based algorithm to detect potential sponge interactions [21]. The algorithm is applicable whenever sequence data is available but does not account for the expression of the RNAs, which provides evidence on the interaction of the RNA species within a given context. Tian et al. [16] find sponge interactions in gastric cancer using microarray expression data. In their approach, they find up- and down-regulated lncRNAs and combine them with miRNA prediction algorithms to construct a lncRNA:miRNA network, but the approach does not consider the correlation structure of RNA expressions. Paci et al. [20] utilizes Pearson correlation and partial correlation analysis to detect sponge interactions in normal and breast cancer samples.

Breast cancer subtypes differ significantly in their molecular profiles and response to therapy. Because miRNAs and mRNAs exhibit different molecular activity patterns in breast cancer subtypes [22–24], it is expected that there will be subtype-specific lncRNA-mediated sponge interactions. Identifying these miRNA sponges can both shed light on the uncharacterized mechanisms of the breast cancer subtypes and potentially help in making better therapeutic decisions. In this work, we use an integrative approach to identify subtype-specific lncRNA:miRNA:mRNA interactions through which lncRNAs compete for binding to shared miRNAs in breast cancer.

To find breast cancer subtype-specific interactions, we systemically analyze lncRNA, miRNA, and mRNA expression profiles of breast cancer patients made available through the Cancer Genome Atlas Project (TCGA) [24]. We first identify statistically related lncRNA:miRNA:mRNA interactions through correlation and partial correlation analysis as in Paci et al. [20] and further refine these candidate interactions using a kernel-based conditional independence test (KCI) [25]. KCI does not assume any parametric form for the random variables that are being tested. Also, for the first time, it is used for finding regulatory interactions. The potential candidate interactions are further filtered in the light of available evidence regarding the miRNA-target interactions. We examine the functional enrichment of mRNAs that participate in sponges, the genomic spatial organization and finally, through the survival analysis of patients, we discover lncRNA-mediated ceRNA interactions with prognostic value.

## Methods

### Data collection and processing

#### lncRNA curation

As lncRNAs are not annotated in TCGA, we curated a list of lncRNAs using GENCODE v24 [26]. Based on GENCODE v24 annotation, 598 of the RNAs present in RNA-Seq expression data are designated as lncRNAs. To minimize erroneous annotations, we further examined each lncRNA’s coding potential with alignment-free method Coding-Potential Assessment Tool (CPAT) [27] and alignment-based method Coding Potential Calculator (CPC) [28]. LncRNAs whose all transcripts are predicted to have high coding potential by both tools are eliminated. The number of lncRNAs that are predicted to have high coding potential by each tool is provided in Figure S1 (Additional file 1).

#### Expression data processing

Level 3 Illumina HiSeq RNA-seq gene expression and miRNA expression data for human breast cancer were collected from The Cancer Genome Atlas [24] on August 9^th^ 2014 (version 1) using the TCGA data portal (https://portal.gdc.cancer.gov/legacy-archive/search/f). The patient survival data were obtained from the UCSC Cancer Genomics Browser on June 31^st^ 2016. Only the patient data that concurrently include mRNA, lncRNA and miRNA expression data were used. Patients were divided into subtypes based on information in TCGA defined by PAM50. The four subtypes used are Luminal A, Luminal B, Basal, HER2. The number of patients in each subtype is provided in Table S1 (Additional file 1).

In expression data, Reads Per Kilobase Million Reads (RPKM) values were used. To eliminate the genes and miRNAs with very low expression, we assumed that RKPM values below 0.05 are missing and filtered out RNAs that are missing in more than 20 of the samples in each subtype. Expression values are log2 transformed. RNAs that do not vary across samples were filtered. We eliminated the genes with the median absolute deviation (MAD) below 0.5.

### Statistical analysis for finding lncRNA mediated ceRNA interactions

To identify ceRNA interactions between lncRNA:miRNA:mRNA, we performed correlation analysis and kernel-based conditional independence test on expression data. Below, random variable *X* denotes the expression level of a lncRNA, random variable *Y* indicates the expression level of a mRNA, and finally random variable *Z* denotes the expression level of a miRNA.

#### Correlation and partial correlation analysis

For a given ceRNA interaction, we expect expression values of the lncRNA and mRNA to be positively correlated, and if this correlation relies on miRNA expression, the correlation between mRNA, and lncRNA should weaken when miRNA expression is taken into account. To quantify this, first Spearman rank order correlation was calculated between lncRNA and mRNAs, which we denote with *ρ*_*lncRNA*,*mRNA*_. Next, we calculated the Spearman partial rank order correlation between lncRNA and mRNA, this time controlling for miRNA expression, *ρ*_*lncRNA*,*mRNA*|*miRNA*_, as follows:

1 $$\begin{array}{@{}rcl@{}} \rho_{X, Y \, \mid \,{Z}} = \frac{\rho_{X, Y} - \rho_{X, Z} \enspace \rho_{Y,Z }}{\sqrt{1 - \rho^{2}_{X, Z}} \sqrt{1 - \rho^{2}_{Y, Z}}} \end{array} $$

The difference between the correlation and the partial correlation for a miRNA measures the extent the miRNA *Z* is effective in the statistical correlation of *X* and *Y*. This value is calculated:

2 $$\begin{array}{@{}rcl@{}} S_{Z} = \rho_{X,Y} - \rho_{X, Y \, \mid \,{Z}} \end{array} $$

As we look for strongly positively correlated lncRNA and mRNA pairs, only those with correlation *ρ*_*X*,*Y*_>0.5 (*p*-value < 0.05) were considered. Among those, RNA triplets where *S*_*Z*_ is larger than a threshold value, *t*, were retained. As it is hard to determine what cutoff is meaningful, we conducted our analysis at two different thresholds, *t*=0.2 and *t*=0.3. *t*=0.2 corresponds to approximately to the 99^*th*^ percentile of the distribution of the *S* (see Additional file 1: Figure S10) and *t*=0.3 is chosen to obtain a even more stringent list.

#### Kernel based conditional independence test

To find lncRNA interactions we also test directly for conditional independence. In a ceRNA interaction, if the interaction of a particular pair of lncRNA (X) and mRNA (Y) were through a shared miRNA (Z), we would expect that lncRNA and mRNA expressions to be conditionally independent given the miRNA expression level. Conditional independence is denoted by *X*⊥⊥*Y*∣*Z*. *X* and *Y* are conditionally independent given *Z* if and only if the **P**(*X* ∣ *Y*,*Z*)=**P**(*X* ∣ *Z*) (or equivalently **P**(*Y* ∣ *X*,*Z*)=**P**(*Y* ∣ *Z*) or **P**(*X*,*Y* | *Z*)=**P**(*X* | *Z*)**P**(*Y* | *Z*)). That is if *X* and *Y* are conditionally independent given *Z*, further knowing the values of *X* (or *Y*) does not provide any additional evidence about *Y*(or *X*).

There are conditional independence tests available for continuous random variables [25, 29–31]. In our work we employ, kernel-based conditional independence (KCI) test proposed by Zhang et al. [25] as it does not make any distributional assumptions on the variables tested. Furthermore, KCI-test does not require explicit estimation of the joint or conditional probability densities and avoids discretization of the continuous random variables, both of which require large sample sizes for an accurate test performance. Below we describe the KCI-test briefly, details of which can be found in [25].

KCI-test defines a test statistic which is calculated from the kernel matrices associated with *X*, *Y* and *Z* random variables. A kernel function takes two input vectors and returns the dot product of the input vectors in a transformed feature space, $k: \mathcal {X} \times \mathcal {X} \rightarrow \mathbb {R}$. The feature transformation is denoted by $\Phi : \mathcal {X} \rightarrow \mathcal {H} $ [32], *k*(**x**_*i*_,**x**_*j*_)=〈*Φ*(**x**_*i*_)·*Φ*(**x**_*j*_)〉. In this work we use the Gaussian kernel, $k\left (\mathbf {{x}}_{i},\mathbf {{x}}_{j} \right) = \exp \left (-\frac {\| \mathbf {{x}}_{i}-\mathbf {{x}}_{j}\|^{2}}{2\sigma ^{2}_{x}}\right)$, where *σ*>0 is the kernel width. CI and KCI are based on kernel matrices of *X*, *Y* and *Z*, which are calculated by evaluating the kernel function for all pairs of samples, i.e. the (i,j)th entry of **K**_*X*_ is *k*(**x**_*i*_,**x**_*j*_). The corresponding centralized kernel matrix is $\tilde {\mathbf {{K}}}_{X} \overset {\Delta }{=} \mathbf {{H}} \mathbf {{K}}_{X} \mathbf {{H}}$ where $\mathbf {{H}} = \mathbf {{I}} - \frac {1}{n}\mathbf {{1}}\mathbf {{1}}^{T}$ where **I** is the *n*×*n* identity matrix and **1** is a vector 1's. $ \tilde {\mathbf {{K}}}_{Y}$ and $\tilde {\mathbf {{K}}}_{Z}$ are similarly calculated for *Y* and *Z* variables.

Given the i.i.d. samples $\textbf {x}\overset {\Delta }{=} (x_{1},x_{2},\ldots,x_{n})$ and $\textbf {y}\overset {\Delta }{=} (y_{1},y_{2},\ldots,y_{n})$, the unconditional kernel test first calculates the centralized kernel matrices, $\tilde {\mathbf {{K}}}_{X}$ and $\tilde {\mathbf {{K}}}_{Y}$ from the samples **x** and **y** and then eigenvalues of the centralized matrices. The eigenvalue decompositions of centralized kernel matrices $\tilde {\mathbf {{K}}}_{X}$ and $\tilde {\mathbf {{K}}}_{Y}$ are $\tilde {\mathbf {{K}}}_{X} = \mathbf {{V}}_{x}\Lambda _{x} \mathbf {{V}}^{T}_{x}$ and $\tilde {\mathbf {{K}}}_{Y} = \mathbf {{V}}_{y}\Lambda _{y}\mathbf {{V}}^{T}_{y}$. Here *Λ*_*x*_ and *Λ*_*y*_ are the diagonal matrices containing the non-negative eigenvalues *λ*_**x**,*i*_ and *λ*_**y**,*i*_ in descending order, respectively. **V**_*x*_ and **V**_*y*_ matrices contain the corresponding eigenvectors. Zhang et al. [25] show that under the null hypothesis that X and Y are independent, the following test statistic:

3 $$\begin{array}{@{}rcl@{}} T_{UI} \overset{\Delta}{=} \frac{1}{n} \text{Tr} \left(\tilde{\mathbf{{K}}}_{X} \tilde{\mathbf{{K}}}_{Y} \right)  \end{array} $$

has the same asymptotic distribution (*n*→*∞*) as

4 $$\begin{array}{@{}rcl@{}} \tilde{T}_{UI} \overset{\Delta}{=} \frac{1}{n^{2}} \sum^{n}_{i,j = 1} \lambda_{\mathbf{{x}},i} \lambda_{\mathbf{{y}},i} z^{2}_{i,j}, \end{array} $$

Here *z*_*i*,*j*_ are i.i.d. standard Gaussian variables, thus $z^{2}_{i,j}$ are i.i.d $\chi _{1}^{2}$ - distributed. The unconditional independence test procedure involves calculating *T*_*UI*_ according to Eq. (3). Empirical null distribution of $\tilde {T}_{UI}$ is simulated by drawing i.i.d random samples for $z^{2}_{i,j}$ variable from $\tilde {\chi }^{2}$. Finally, the *p*-value is calculated by locating *T*_*UI*_ in the empirical null distribution.

The kernel conditional independence test also makes use of the centralized kernel matrices. Under the null hypothesis that *X* and *Y* are conditionally independent given *Z*, the following test statistic is calculated:

5 $$\begin{array}{@{}rcl@{}} \tilde{T}_{CI} \overset{\Delta}{=} \frac{1}{n} \text{Tr} \left(\tilde{\mathbf{{K}}}_{\ddot{X}\, \mid \,{Z}} \tilde{\mathbf{{K}}}_{Y \, \mid \,{Z}} \right), \end{array} $$

where $\ddot {X} \overset {\Delta }{=} (X,Z)$ and $\mathbf {{K}}_{\ddot {X}}$ is the centralized kernel matrix for $\ddot {X}$. As Zhang et al. [25] report has the same asymptotic distribution as

6 $$\begin{array}{@{}rcl@{}} \tilde{T}_{CI} \overset{\Delta}{=} \frac{1}{n} \sum^{n^{2}}_{k = 1} \mathring{\lambda_{k}} \cdot z^{2}_{k} \end{array} $$

The details of the definition of *λ*_*k*_ ¨ and $z^{2}_{k}$ can be found in [25]. The procedure involves calculating the empirical *p*-value based on the test statistic as defined in Eq. (3) and simulating the null distribution based on Eq. (6).

Using the unconditional kernel independence test, we first test the null hypothesis that a lncRNA and mRNA pair is independent. We consider pairs for whom the null hypothesis is rejected at the 0.01 significance level. For each of the lncRNA:mRNA pair, we test their conditional independence given each miRNA separately using KCI. The triplet where the lncRNA:mRNA pair given an miRNA is found to be independent at significance level 0.01 are considered as potential lncRNA-mediated ceRNAs.

### Filtering ceRNAs based on miRNA-target interactions

To identify interactions that are biologically meaningful, we filtered the potential ceRNA interactions that were not supported by miRNA target information. The miRNA:mRNA and miRNA:lncRNA interactions are retrieved from multiple databases as listed in Table 1. The candidate sponges are retained if both mRNA and lncRNA have support for being targeted by the miRNA of the sponge.

**Table 1:** Computationally and experimentally validated miRNA-target databases used for mRNA and lncRNA

miRNA-Target Databases	mRNA	lncRNA	P/C	Reference
TargetScan	+	+	P	[56]
miRcode	+	+	P	[57]
mirSVR	+	+	P	[58]
PITA	+	+	P	[59]
RNA22	+	+	P	[60]
lnCeDB		+	P	[61]
mirTarBase	+	+	E	[62]
Diana LncBase		+	E	[63]

Plus signs denote databases that are used for the miRNA interactions of the RNA type. ‘P’ denotes predicted target information while ‘E’ denotes experimentally supported target information

### Identifying ceRNAs with prognostic value

To evaluate the ceRNA interactions in terms of their prognostic potential, we analyzed the survival of the patients based on the expression patterns of each sponge interaction. In a sponge interaction, we expect the lncRNA and mRNA to be regulated in the same direction and miRNA to be in the opposite direction. For each ceRNA found in a subtype, the patients are divided into two groups based on the regulation patterns of the RNAs that participate in the ceRNA. For the up-down-up pattern, the first group comprises patients whose sponge lncRNA and mRNA are up-regulated, and miRNA is down-regulated; the second group includes all patients that do not fit in this pattern. Similarly, we divide the patients based on the down-up-down pattern: if both lncRNA and mRNA are down-regulated whereas miRNA is unregulated, such patients constitute one group, and the rest of the patients constitute the second group.

Based on this grouping, we tested whether the ceRNA expression pattern can divide the patients into two groups, where the survival distribution of the groups are different using log-rank test [33] (*p*-value < 0.05). Note that since the log-rank test is not reliable when one of the group sizes is small, we only consider the cases where after dividing the patients based on the expression pattern, the group sizes are larger than 10. Since the observed significant difference could be due to a single RNA molecule prognostic value, we only considered ceRNAs as prognostic if none of the RNAs can by itself divide the patients into groups that differ in terms of their survival distributions significantly. In each subtype, we split the patients as up-regulated and down-regulated for each of the RNA participating in the ceRNA interaction separately. If at least one of the molecules leads to groups with significant survival difference (log-rank test, *p*-value < 0.05), we disregard this ceRNA from the list of prognostic ceRNAs. This last step ensures that the prognostic difference is due to the interactions between the RNAs but not stem from the expression of the single RNA's expression patterns. We also tested whether RNAs were prognostic in other subtypes by conducting the log-rank test on expression data of the RNAs in other subtypes.

The identified prognostic interactions’ are further summarized with *f*-score that reflects the interaction’s prognostic value compared to the most prognostic RNA of the interaction.

7 $$ f_{xyz}= - \log \frac{p_{xyz}} {\texttt{min} (p_{x}, p_{y}, p_{z})}  $$

Here, *p*_*xyz*_ is the *p*-value attained in testing whether patient survivals differ based on the log-rank test of the triplet whereas *p*_*x*_, *p*_*y*_, *p*_*z*_ indicate the *p*-values obtained by testing patient survival distribution differences due to lncRNA, mRNA, and miRNA expression patterns, respectively. Thus *f*-score is the log-fold decrease in the *p*-value when the patients are divided based on the interaction.

In the above analysis, RNAs that have expression levels above (or below) a particular threshold value are considered up-regulated (or down-regulated). This threshold value is selected among the candidate cut-off values of expression as the one that results in the lowest *p*-value in the log-rank test when patients are divided based on this cut-off. The candidate cut-off values are the 10^th^ and 90^th^ percentiles, mean, median or the lower and upper quartiles of the expression values of the patients in each subtype.

### Pathway and GO enrichment analysis

**Fig. 1:**
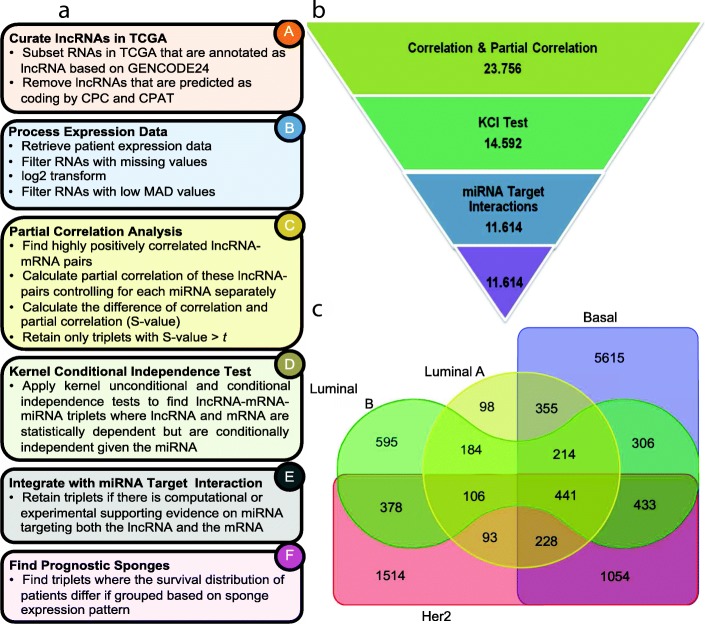
**a** Overview of the methodology, each box represents a step in the methodology. Steps B-F are conducted for each breast cancer subtype separately. **b** The number of ceRNAs remained after each main filtering step when *t*=0.2 (Step C in Fig. 1**a**). **c** Venn diagram of ceRNA interactions discovered in each of the breast cancer molecular subtype

We conducted and GO enrichment of mRNAs that participate in subtype-specific sponges. Enrichment tests are conducted with clusterProfiler [34] with Bonferroni multiple hypothesis test correction. In deciding enriched pathways and GO terms, a *p*-value cut-off of 0.05 and FDR cut-off of 1×10^−4^ are used. In both pathway and GO enrichment analyses, the background genes were the union of mRNAs that remained after the MAD filtering step (Step B in Fig. 1a). For pathway enrichment analysis, different pathway data sources were downloaded from Baderlab GeneSets Collection [35]. List of all pathways that are employed in this analysis is provided in Table S2 (Additional file 1). Redundant pathways are eliminated when different sources are combined. Additionally, a pathway enrichment analysis is conducted with KEGG pathways (downloaded on February 28^th^ 2017).

### Clustering mRNAs

If mRNAs are highly correlated with each other, we often find that correlated mRNAs participate in ceRNA interactions with the lncRNA and miRNA pair.

We consider the mRNAs that participate in a ceRNA interaction with the same pair of lncRNA and miRNA. If all mRNAs are strongly correlated among each other, where all the pairwise correlations are above 0.7, all mRNAs are assigned into the same cluster. Otherwise, we apply Ward hierarchical clustering method to find groups of correlated mRNAs [36]. We determine the optimal number of clusters with Mojena’s stopping rule [37] using Milligan and Cooper’s [38] correction.

## Results

### Overview of discovered ceRNA interactions

The ceRNA hypothesis states that transcripts with shared miRNA binding sites compete for post-transcriptional control [12, 15]. Based on this hypothesis, we set out to discover subtype-specific breast cancer ceRNA interactions where lncRNAs can act as miRNA sponges to reduce the amount of miRNAs available to target mRNAs. We employ the methodology summarized in Fig. 1a and identify ceRNAs specific to four molecular subtypes of breast cancer: Luminal A, Luminal B, HER2, and Basal. The number of candidate ceRNA interactions that remain after each main step when in the partial correlation analysis step *S* value threshold *t*=0.2 is employed, is provided in Fig. 1b (see Figure S2(A) in Additional file 1 for *t*=0.3). The total number of ceRNA interactions found in all subtypes is 11,614. Figure 1c shows the Venn diagram of number of ceRNA interactions discovered for the four subtypes (see Figure S2(B) in Additional file 1 for *t*=0.3). Although there are sponges that are detected in multiple subtypes, there are also a large number of sponges that are only specific to a single subtype (Table S3 and Figures S3A and S3B in Additional file 1). The list of sponges identified in each subtype, their partial correlation analysis, KCI-test results and target information are provided in Additional file 2.

**Fig. 2:**
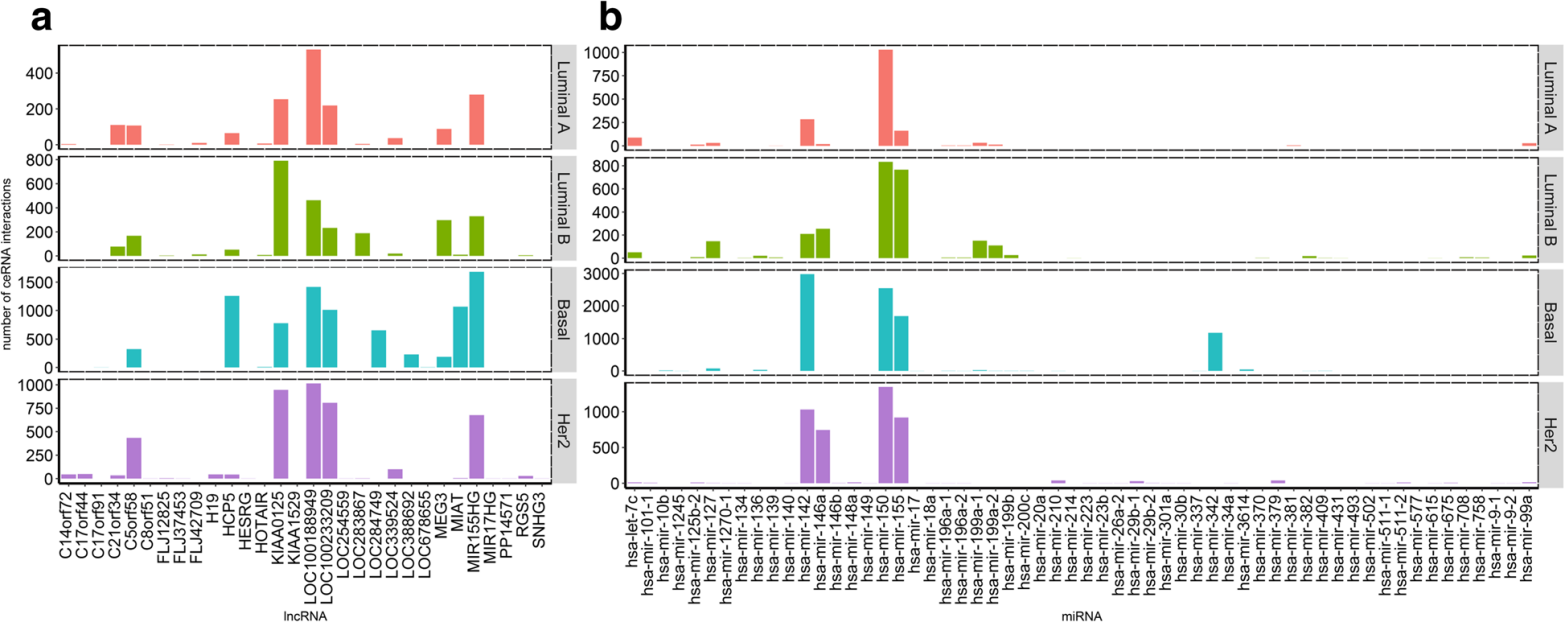
Number of ceRNA interactions discovered that **a** lncRNAs and **b** miRNAs take part in each breast cancer subtype (*t*=0.2)

We analyze the specificity of the individual RNAs that participate in each of the subtypes. Figures 2a and b display the number of sponges per lncRNA and miRNA for *t*=0.2 (Figure S3C in Additional file 1 for *t*=0.3). Some lncRNAs and miRNAs participate in sponges of all the subtypes (Table 2); i.e., KIAA0125 (FAM30A) participates in a large number of sponges across the four subtypes. KIAA0125 has been reported to act as an oncogene in bladder cancer related to cell migration and invasion [39]; however, no functional relevance to breast cancer has been reported to date. HOTAIR, which is one of the lncRNAs that has been associated with metastasis [40], is found to participate in sponges of all the subtypes except HER2. Similarly, miRNAs hsa-miR-142, hsa-miR-150, and hsa-miR-155 participate in ceRNA interactions of all subtypes.

**Table 2:** List of lncRNAs & miRNAs that are found to participate in sponges of all four subtypes

miRNA	lncRNA
hsa-miR-142	LOC100188949
hsa-miR-196a-1	C5orf58
hsa-miR-127	LOC100233209
hsa-miR-155	HCP5
hsa-miR-150	KIAA0125
hsa-miR-196a-2	C21orf34
hsa-miR-125b-2	MIR155HG

There are also RNAs that take part in sponges exclusively in a single subtype (Table S4 in Additional file 1). For example, the lncRNA C17orf44 (LINC00324) is specific to HER2 (Fig. 2a) while hsa-miR-342 is only found in Basal ceRNA interactions (Fig. 2b). Several studies indicated that miR-342 is linked to BRCA1 mutated breast cancer, most of which are the Basal subtype [41–43]. Similarly, some mRNAs are involved in ceRNA interactions only in a single subtype (see Additional file 3 for all the mRNAs in the interactions and see for only the prognostic mRNAs see Additional file 4). These subtype-specific RNAs are of great value for understanding the dysregulated cellular mechanisms in each subtype.

The lncRNA:mRNA networks for each subtype are shown in Figure S4 (Additional file 1) and Additional file 5. In these networks, each node denotes a lncRNA or an mRNA while an edge represents an interaction through a shared miRNA. The number of nodes and edges are provided in Table S5 (Additional file 1). In Luminal A, lncRNA LOC100188949 (LINC00426) regulates the majority of the sponge interactions, while C21orf34 (MIRHG99AHG) also form a smaller connected component of its own. In Luminal B, KIAA0125 is at the center of the many interactions while a few other lncRNAs among them are HOTAIR and C21orf34 mediates a small number of interactions. Basal and HER2 subtypes include a large number of interactions. In Basal subtype, among others HCP5, MIR155HG, MIAT are the hubs of the network. In HER2 subtype KIAA0125, LOC100188949 and LOC100233209 (PCED1B-AS1) are the top 3 largest hubs.

We find that the same lncRNA:miRNA pair participates in multiple sponge interaction with different mRNAs. As an example, HER2 subtype-specific C14orf72:hsa-miR-150 lncRNA:miRNA pair interacts with 45 different mRNAs, the same is not true for lncRNA:mRNA pairs. The number of ceRNA interactions per lncRNA:miRNA pairs is provided in Figure S5 (Additional file 1). We also analyze the data by clustering mRNAs that participate in a sponge with the same lncRNA:miRNA pair based on mRNA expression correlation. The list of the identified sponges in the view of these clusters are provided in Additional file 6.

### Spatially proximal ceRNAs interactions

**Fig. 3:**
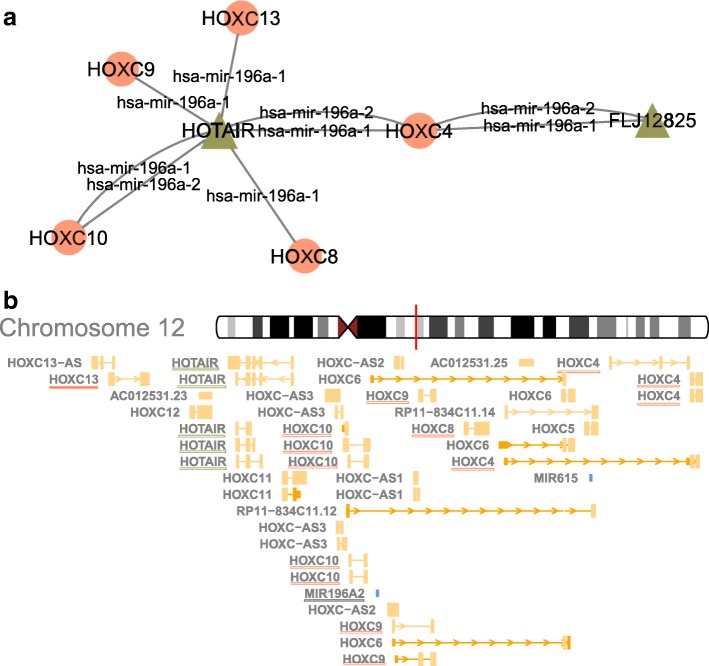
**a** The network of sponge interactions between HOTAIR, hsa-miR-196a miRNAs and HOXC genes. The circles denote lncRNAs, and triangles denote mRNAs. An edge exists between a lncRNA and an mRNA if there is a sponge interaction between them; the edge label indicates the miRNA that regulates the interaction. **b** The genomic locations of the sponge interactions on chromosome 12. Spatially proximal genes are underlined with the same color code of used in (**a**)

In the prior section, we analyzed all possible ceRNA interactions including both distal and spatially proximal ceRNA interactions. Although the regulatory interactions can take place between molecules encoded in different chromosomes, spatial proximity often hints at a tight regulatory coordination. Also there are several studies highlighting the functional relevance of spatially proximal RNA interactions (not necessarily to be a ceRNA interaction) (1, 2), and we reasoned that chromosomal proximity of RNAs involved in a ceRNA interaction could also be functional. Therefore, we analyzed all the ceRNA participating RNAs within 100KB distance of each other, and identified several potentially important proximal ceRNA interactions. For example, we found that on chromosome 12, there is a potential sponge interaction that takes place between HOTAIR, hsa-miR-196a and HOXC genes (Fig. 3a) which could be an important ceRNA interaction to contribute to the HOTAIR’s known oncogenic functions as reported previously.

HOX genes are highly conserved transcription factors that take master regulatory roles in numerous cellular processes including development, apoptosis, receptor signaling, differentiation, motility, and angiogenesis. Their aberrant expression has been reported in multiple cancer types [44]. HOXA is reported to have altered expression in breast and ovarian cancers; other HOX genes are also associated with multiple tumor types, including colon, lung, and prostate cancer. The lncRNA partner of this sponge interaction is HOTAIR. Up-regulation of HOTAIR is associated with metastatic progression and low survival rates in breast, colon, and liver cancer patients [14, 16, 17, 19, 39, 45–48]. The complete list of sponge interactions whose members exhibit such spatial proximity at least between two RNAs in the sponge is provided in Additional file 7. Please note that, whether it is spatially proximal or distal, all the ceRNA interactions are expected to occur in the cytoplasm as the RISC complex necessary for miRNA binding is localized in the cytoplasm.

### Functional enrichment analysis of mRNAs in ceRNAs

To understand the patterns of pathways related to identified sponges, we conducted pathway enrichment of mRNAs that participate in the sponges separately. The top enriched pathways are found to be common across subtypes (see Additional file 1: Figures S6–S7) and these pathways are mostly related to the immune system and signaling pathways, which are essential modulators of cancer progression and therapy response [49]. Interestingly, interferon alpha/beta signaling pathway is among the top pathways for Basal subtype (*p*-value 7.20×10^−23^) while it is not found enriched in other subtypes (*p*-value cut-off 0.05 and FDR cutoff 1×10^−4^).

Considering the key role of interferon signaling in the immune system, and the positive correlation between immune cell infiltration and aggressiveness of Basal subtype of breast cancer, our results suggest that mRNAs involved in ceRNA interactions might contribute to the different immune profile of the Basal subtype [50, 51]. Complement cascade induces cell proliferation which causes carcinogenesis including invasion, cell death, and metastasis [52], which are Basal subtype characteristics. We detected C2, C3, C3AR1, C4A, C7 complement genes in Basal ceRNA interactions. Consequently, complement cascade pathway may be significant for the Basal subtype.

The overlap between the enriched pathways in different subtypes is shown on a Venn diagram (Figure S8 in Additional file 1). The list of pathways that are found enriched only in a single subtype is listed in Table S7 in Additional file 1 with p-value cut-off 0.05 and FDR cutoff 1×10^−4^. Interestingly, the PI3K pathway is found to be enriched specifically in Luminal A. This is interesting as the most frequently mutated gene in Luminal A is PIK3CA (45% of the patients in TCGA), and there are PIK3CA mutations that are specific to this subtype [24]. This suggests that ceRNA interactions might be key regulators of the PI3K pathway, especially in this subtype of tumors which comprises of more than 60% of breast cancers.

Integrin signaling is widely studied in breast cancer literature since integrins incorporate breast cancer progression [53]. Moreover, integrins play key roles in migration, invasion, and metastasis of cancer cells. Enrichment of integring signaling in mRNAs involved in ceRNA networks might suggest that HER2-specific ceRNA interactions might contribute the aggressive progression of HER2 subtype, similar to the Basal subtype of breast cancer.Thus, they drive tumor cell to metastasis [53]. HER2 subtype-specific enriched pathways contain integrin signaling pathways (Table S7 in Additional file 1).

### Prognostic sponge interactions

**Fig. 4:**
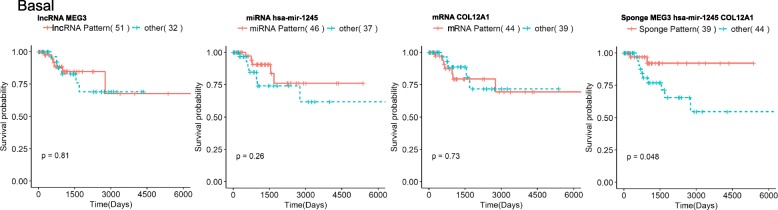
Kaplan-Meir survival plots when patients are divided based on individual expression patterns of the RNAs (the first three plots in each panel) and when patients are divided based on the sponge expression pattern (4th plot) for MEG3, hsa-miR-1245, COL12A1 sponge

**Fig. 5:**
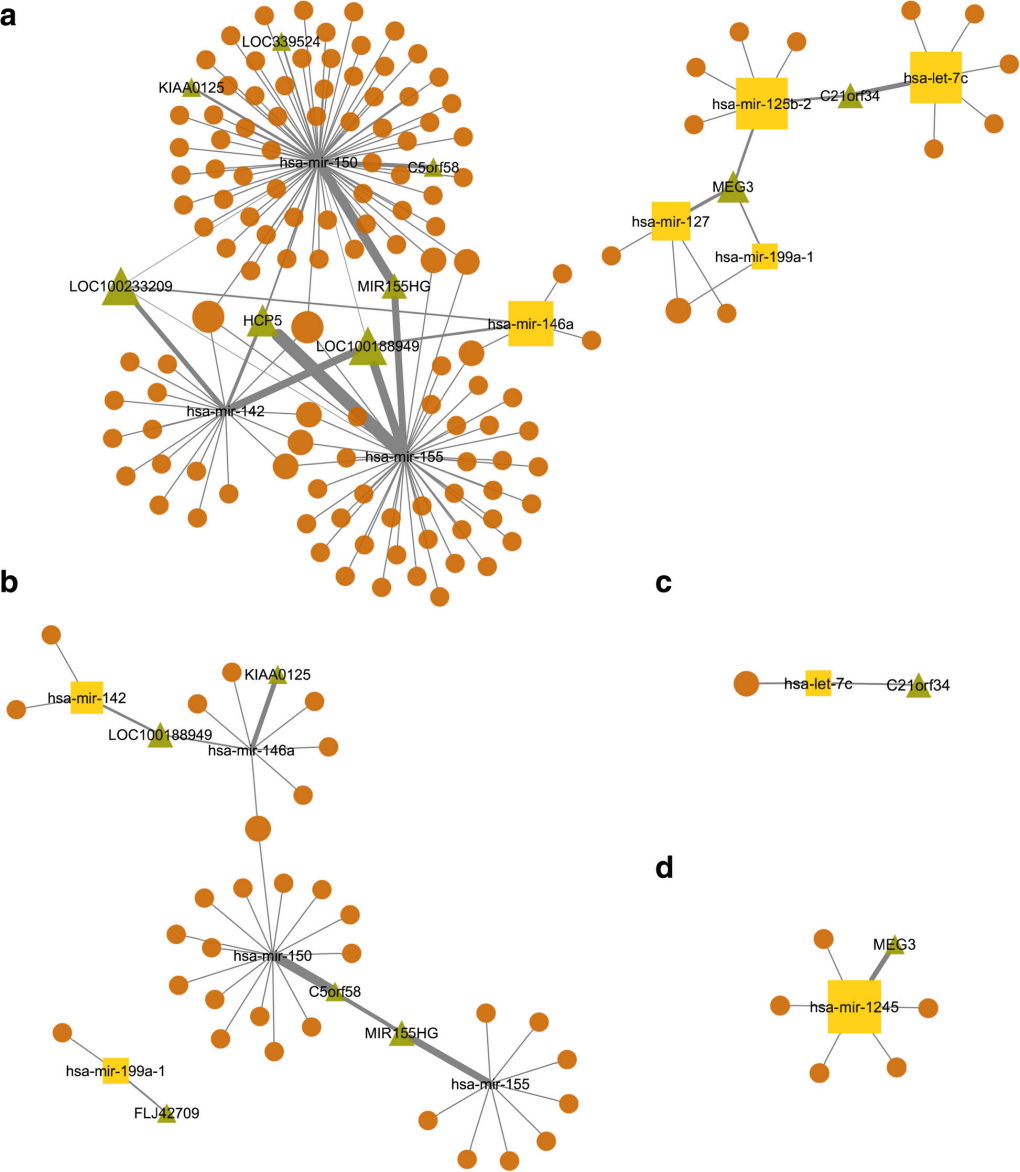
lncRNA:miRNA:mRNA network for all breast cancer subtypes, Luminal A (**a**), Luminal B (**b**), HER2 (**c**), Basal (**d**). lncRNAs are represented by the green triangle symbol, mRNAs are represented by orange ellipse symbol and miRNAs are with the yellow rectangle. Each node size is scaled by its degree, the number of edges incident to the nodes and edge width is scaled by the number of occurrence of the node pair. The network was constructed using the Cytoscape (v3.4.0) [55]

To identify ceRNA interactions with prognostic value in each subtype, for each of the identified sponge we checked whether the sponge expression pattern divides the patients into groups that differ in their survival probability. To this end, for each potential ceRNA based on the participants up or down-regulation pattern, we divided the patients into two groups and checked if the survival of these groups differs significantly using log-rank test (details provided in the Methods section). For the cases, where we observe a significant difference, we further checked if the observed difference could be attributed to the prognostic power of a single RNA molecule in the interaction by performing a log-rank test on each of the constituents’ expression pattern. We only considered the ceRNA interactions as prognostic for this subtype if there was a significant difference in survival when patients were grouped based on ceRNA expression pattern but there was no significant difference if the patients were grouped based on a single RNA molecules’ expression pattern. An example prognostic ceRNA interaction is shown in Fig. 4; patients with a sponge pattern where lncRNA MEG3 and mRNA COL12A1 are high while miRNA miR-1245 low have better survival than other patients (Fig. 4d) while none of the three RNA molecules can separate the patients into groups that differ in survival probabilities individually (Fig. 4a, b and c). This result suggests that examining sponge patterns might have a better prognostic value than that of the individual genes. The networks of prognostic sponges in each subtype highlight that some of the lncRNAs and miRNAs are central in these interactions (Fig. 5 and the Cytoscape file in Additional file 5).

We summarized the prognostic ceRNA interactions’ prognostic values with *f*-score (details in Methods), which is the log-fold decrease in the *p*-value of the ceRNA interaction compared to the best RNA molecule in the ceRNA interaction. The list of prognostic ceRNAs ranked based on the *f*-score is provided in Additional file 8 together with *p*-values of all the log-rank tests conducted. The overall distribution of *f*-scores are provided in Figure S11 (Additional file 1). The presence of each RNA as a participant of a prognostic RNA across each subtype are provided in demonstrated in Figures S9 a, b and c in Additional file 1.

## Discussion

To find the triplets of lncRNA, mRNA, and miRNAs that are likely to form sponge interactions, we develop a method that uses statistical tests on patient RNA expression profiles. We start this analysis with likely physically interacting list of miRNA interactions. To this end, we retrieve experimentally validated and computationally predicted miRNA:lncRNA and miRNA:mRNA information from multiple databases. Due to the limited knowledge on experimentally confirmed interactions, we also choose to include predicted targets in our analysis. While the inclusion of predicted RNA interactions allows us to investigate a wider list of candidates it is also likely to increase the false positive rates. If more experimental target information becomes available, the framework can be altered to exclusively use the experimentally identified miRNA target information and the statistical tests can be performed only on this set of candidate triplets.

A statistical interaction does not automatically imply a physical interaction in the cell, because just like any other intracellular interaction, the RNA-RNA interactions are context dependent. Our predicted interaction set, therefore, constitutes a candidate list that can be probed and tested experimentally. To assist in prioritizing candidate interactions, we curate additional information such as the number of databases supporting a predicted interaction and the number of miRNA binding sites in the lncRNA partner. The latter is important because for the lncRNA to be tittered down by the miRNA, the presence of multiple miRNA binding sites might be required. We also provide subsets of interactions through computational means such as the cis-RNAs interactions and the interactions with prognostic potential. This additional information and the subsets can be used to prioritize interactions for experimental validation and can help explore different aspects of RNA regularity mechanisms in breast cancer subtypes.

One challenge is the unavailability of experimentally validated and lncRNA mediated sponge interactions for breast cancer subtypes. This limits the efforts to assess the statistical power and the false positive rate of our method and complicates the choice of cut-off values used in the compilation of the final candidate list. For this reason, we report our results at two cut-off values that differ in their stringency. To assist with these analyses, we also perform several additional analyses to investigate the relevance of the discovered potential interactions. This way we can validate the interaction list with indirect supporting evidence. Firstly, a functional enrichment analysis of mRNAs that involve subtype-specific sponges is done. This analysis reveals subtype specific mRNA partners that are enriched with pathways/processes known to be specific to some of the subtypes. We consider this as an indirect validation. Secondly, the spatial organization of the RNA triplets that participate in the genome reveals that some of the sponges are positioned in close proximity of each other on the genome, hinting a regulatory relationship between these RNAs. Thirdly, a subset of the interactions is found to have prognostic value. Based on the sponge expression patterns, patients can be divided into two groups that differ in terms of their survivals.

## Conclusion

As transcriptome is cataloged with greater depth, it has become evident that the vast majority of the mammalian transcriptome is non-coding. One type of non-coding RNAs is lncRNA. A growing body of evidence demonstrates that lncRNAs are deregulated in cancer just like mRNAs and miRNAs [54]. For example, over-expression of the lncRNA HOTAIR in breast cancer patients is reported to be highly predictive of patient survival and progression to metastasis [40]. An emerging role of lncRNAs is that they compete for binding to miRNAs, acting as a sponge to regulate the gene activity. This three-way regulatory interaction between lncRNAs, miRNAs, and the mRNAs is observed in multiple cancer types, including breast cancer. Our contribution in this work is two folds. Firstly, we identify potential subtype-specific lncRNA mediated sponge interactions in breast cancer. These findings can be probed and tested by experimental analyses and potentially help uncover unknown molecular mechanisms of breast cancer subtypes. Secondly, to achieve this analysis, we develop an integrative methodology, which has broader applicability and relevance to studies on other diseases or analyses on normal cell.

## Additional files

Additional file 1 Supplementary text file that contains additional figures and tables. (PDF 2898 kb)

Additional file 2 List of Subtype Specific lncRNA mediated Sponge Interactions. Partial correlation and KCI values are provided in this list. Moreover, target interaction for lncRNA:miRNA and mRNA:miRNA are given with their supported databases. (XLSX 2705 kb)

Additional file 3 List of mRNAs participating in sponge interactions and their distribution over subtypes. (XLSX 70 kb)

Additional file 4 List of mRNAs participating in prognostic sponge interactions and their distribution over subtypes. (XLSX 18 kb)

Additional file 5 Prognostic Sponge Interactions Cytoscape Network File. (CYS 46 kb)

Additional file 6 List of mRNA clusters in sponge interactions. For the same subtype and same lncRNA:miRNA interactions mRNAs clustered. (XLSX 89 kb)

Additional file 7 List of Spatially Proximal Sponge Interactions. Chromosome locations of the RNAs are given. Moreover, how many of the RNAs in the sponge(lncRNA, mRNA or/and miRNA) are spatially proximal are provided in this list. (XLSX 93 kb)

Additional file 8 List of Prognostic Sponge Interactions, its corresponding pvalues and number of patients in groups are provided. (XLSX 2108 kb)

## Abbreviations

ceRNA: competing endogenous RNA
CPAT: Coding-potential assessment tool
CPC: Coding potential calculator
KCI: Kernel-based conditional independence
lncRNA: long non-coding RNA
MAD: Median absolute deviation
miRNA: microRNA
mRNA: messenger RNA
ncRNA: non-coding RNA
TCGA: The cancer genome atlas project
RPKM: Reads per Kilobase million reads
