# Supplementary material for: Discovering lncRNA mediated sponge interactions in breast cancer molecular subtypes
Source: BMC Genomics. 2018 Sep 4;19:650. doi: 10.1186/s12864-018-5006-1 (PMC6122485; doi:10.1186/s12864-018-5006-1)
Supplement: Supplementary file 1 — Supplementary text file that contains additional figures and tables. (PDF 2898 kb) [file 12864_2018_5006_MOESM1_ESM.pdf]

Supplementary Materials for ‘Discovering lncRNA Mediated Sponge Interactions in Breast Cancer Molecular Subtypes’

Gulden Olgun[1], Ozgur Sahin[2], Oznur Tastan[3*]
[1] Department of Computer Engineering, Bilkent University, Ankara, Turkey
[2] Department of Molecular Biology and Genetics, Faculty of Science, Bilkent University, Ankara, Turkey
[3] Faculty of Engineering and Natural Sciences, Sabanci University, Tuzla, Istanbul, Turkey
* otastan@sabanciuniv.edu

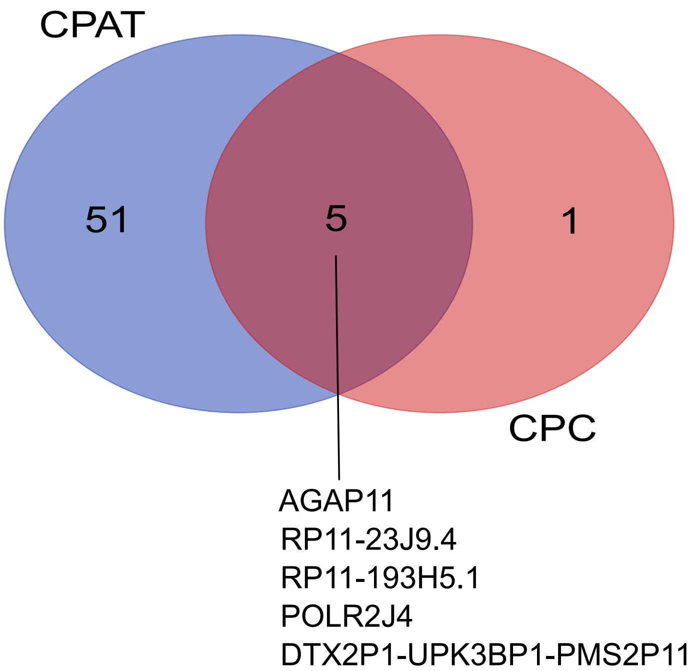

Figure S1: Venn diagrams for lncRNAs that are found coding with CPAT [1] and/or CPC [2].

Table S1: Number of patients in each breast cancer subtype.

	**# of Patients**	
**Subtypes**	**Expression Data**	**Clinical Data**
Luminal A	211	207
Luminal B	112	110
Basal	85	83
HER2	54	50

Table S2: Pathway data sources utilized for enrichment analysis and number of pathways in each data source.

Source	# of Pathways	Version/Frozen Date
HumanCyc	240	v20.5
Institute of Bioinformatics (IOB)	33	July 2011
MSigdb	520	v5.1
NCI	223	Feb 2016
NetPath	25	Jun 2016
Panther	175	Jul 2016
Reactome	18889	Dec 2016

Table S3: Number of ceRNA interactions identified in each breast cancer subtypes at $t = 0.3$ and $t = 0.2$. Total number of all ceRNA interactions and number of subtype specific ceRNAs are provided.

	# of ceRNA Interaction			
**Subtypes**	**0.3 Threshold**		**0.2 Threshold**	
	Found All	Subtype Specific	Found All	Subtype Specific
**Luminal A**	57	22	1719	98
**Luminal B**	124	51	2657	595
**Basal**	1479	1309	8646	5615
**HER2**	535	371	4247	1514

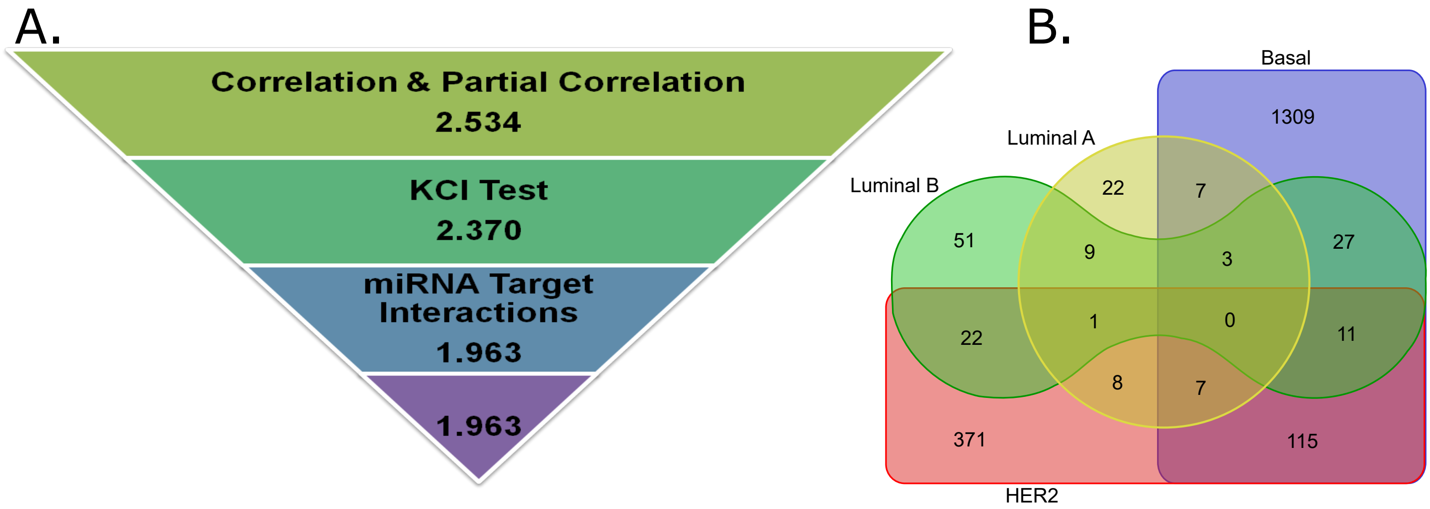

Figure S2: A) Number of ceRNAs remained after each main filtering step when $t = 0.3$ threshold is used. B) Venn diagram of ceRNA interactions discovered in each of the breast cancer molecular subtype.

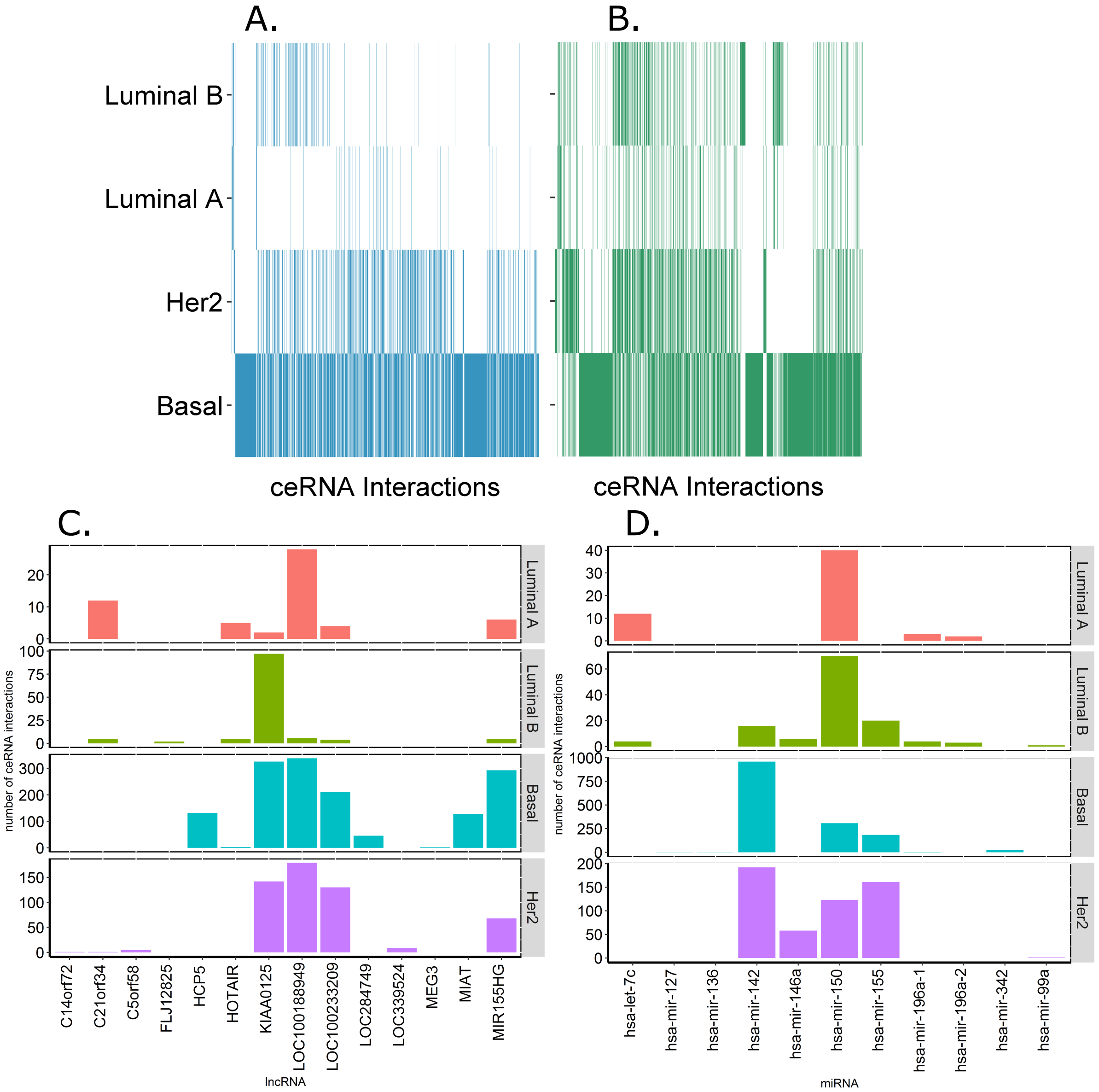

Figure S3: A-B) Heatmaps display the distribution of ceRNAs over the subtypes for A) $t = 0.3$ and B)$t = 0.2$. Blue and green cells indicate the ceRNA interaction is discovered in the given subtype, while white color indicates it is not. Number of ceRNA interactions discovered that per C) each lncRNA and per B) each miRNA in each breast cancer subtypes ($t = 0.3$).

Table S4: List of lncRNAs & miRNAs that are found in sponges of single subtype.

Subtype	miRNA	lncRNA
Luminal A	hsa-miR-381	
Luminal B	hsa-miR-431	
	hsa-miR-758	
	hsa-miR-708	
	hsa-miR-214	
	hsa-miR-370	
HER2	hsa-miR-29b-1	FLJ37453
	hsa-miR-140	MIR17HG
	hsa-miR-149	C17orf44
	hsa-miR-23b	LOC254559
	hsa-miR-9-1	C8orf51
	hsa-miR-379	PP14571
	hsa-miR-675	H19
	hsa-miR-101-1	SNHG3
	hsa-miR-502	HESRG
	hsa-miR-30b	
	hsa-miR-223	
	hsa-miR-34a	
	hsa-miR-26a-2	
	hsa-miR-9-2	
	hsa-miR-511-1	
	hsa-miR-1270-1	
	hsa-miR-148a	
	hsa-miR-146b	
	hsa-miR-18a	
	hsa-miR-29b-2	
	hsa-miR-301a	
Basal	hsa-miR-10b	LOC284749
	hsa-miR-1245	C17orf91
	hsa-miR-493	LOC388692
	hsa-miR-342	KIAA1529
	hsa-miR-17	LOC678655
	hsa-miR-20a	
	hsa-miR-577	
	hsa-miR-337	
	hsa-miR-3614	
	hsa-miR-200c	

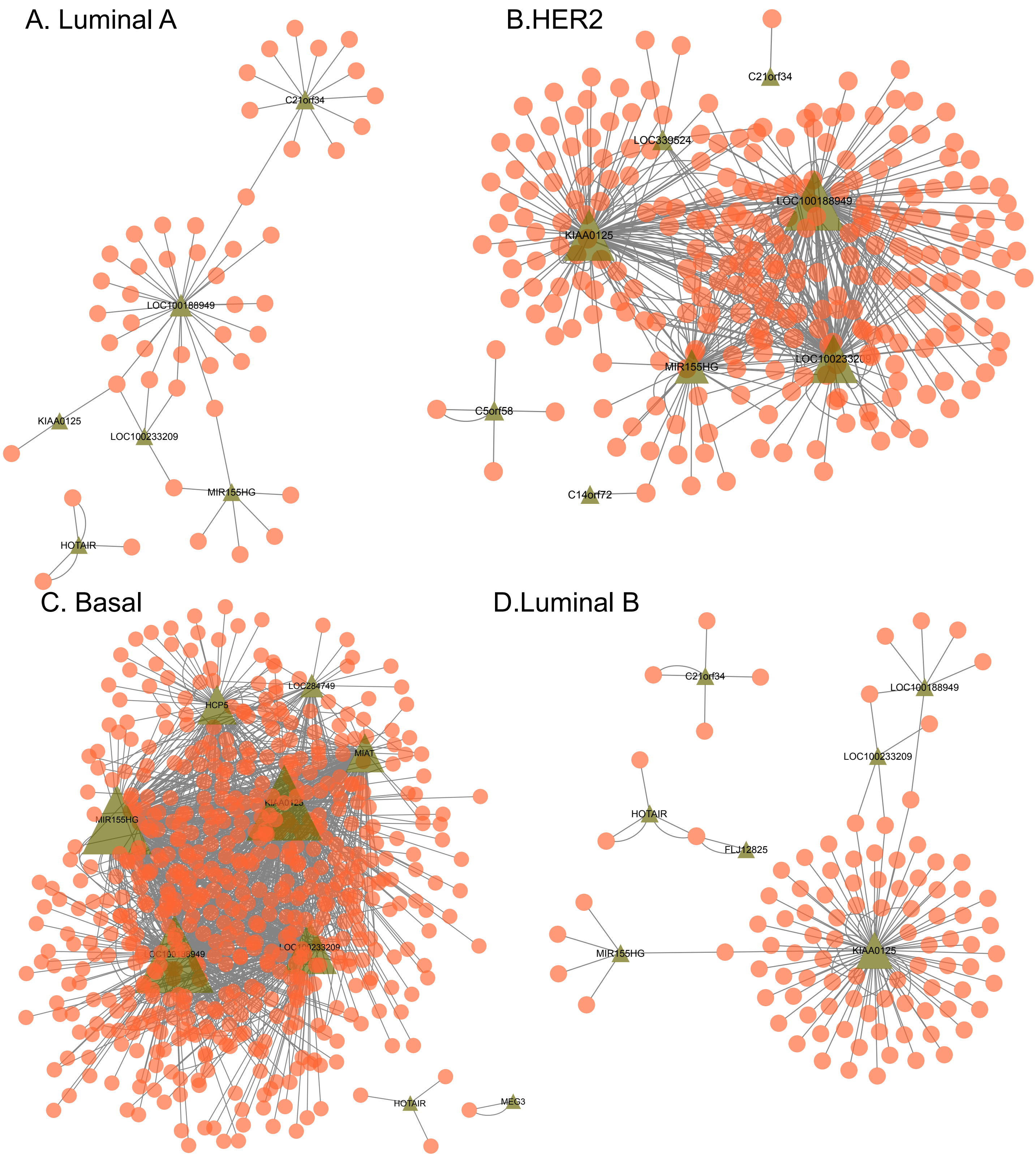

Figure S4: lncRNA-mRNA network for each breast cancer subtypes. Green triangle nodes represent lncRNA and circle orange nodes represents mRNA. An edge between an mRNA and a lncRNA is drawn to represent a ceRNA interaction through a miRNA. Node size is in proportion to degree of the node. The network plot was generated with Cytoscape(v3.4.0)[3].

Table S5: Number nodes and edges for bipartite lncRNA-mRNA networks for each breast cancer subtypes where each node denotes lncRNA or mRNA and each edge represents a lncRNA-mRNA interaction,miRNA.

Subtypes	# of Nodes	# of Edges
Luminal A	54	57
Luminal B	106	124
Basal	574	1479
HER2	272	535

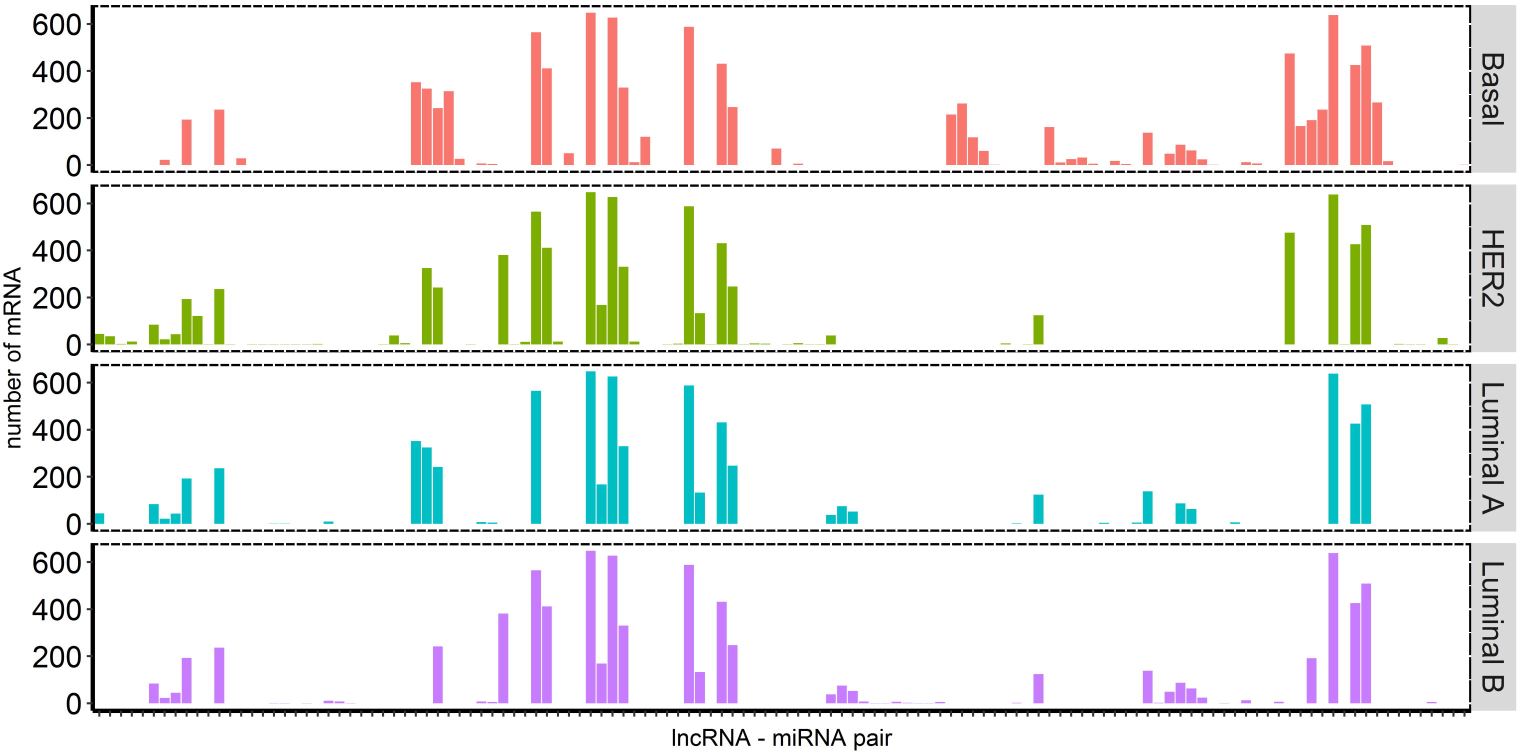

Figure S5: Number of mRNAs that each lncRNA-miRNA pair interacts with in each subtype ($t = 0.2$).

Table S6: Number of unique lncRNA - miRNA

Subtypes	# of lncRNA-miRNA pair
Luminal A	37
Luminal B	52
HER2	64
Basal	60

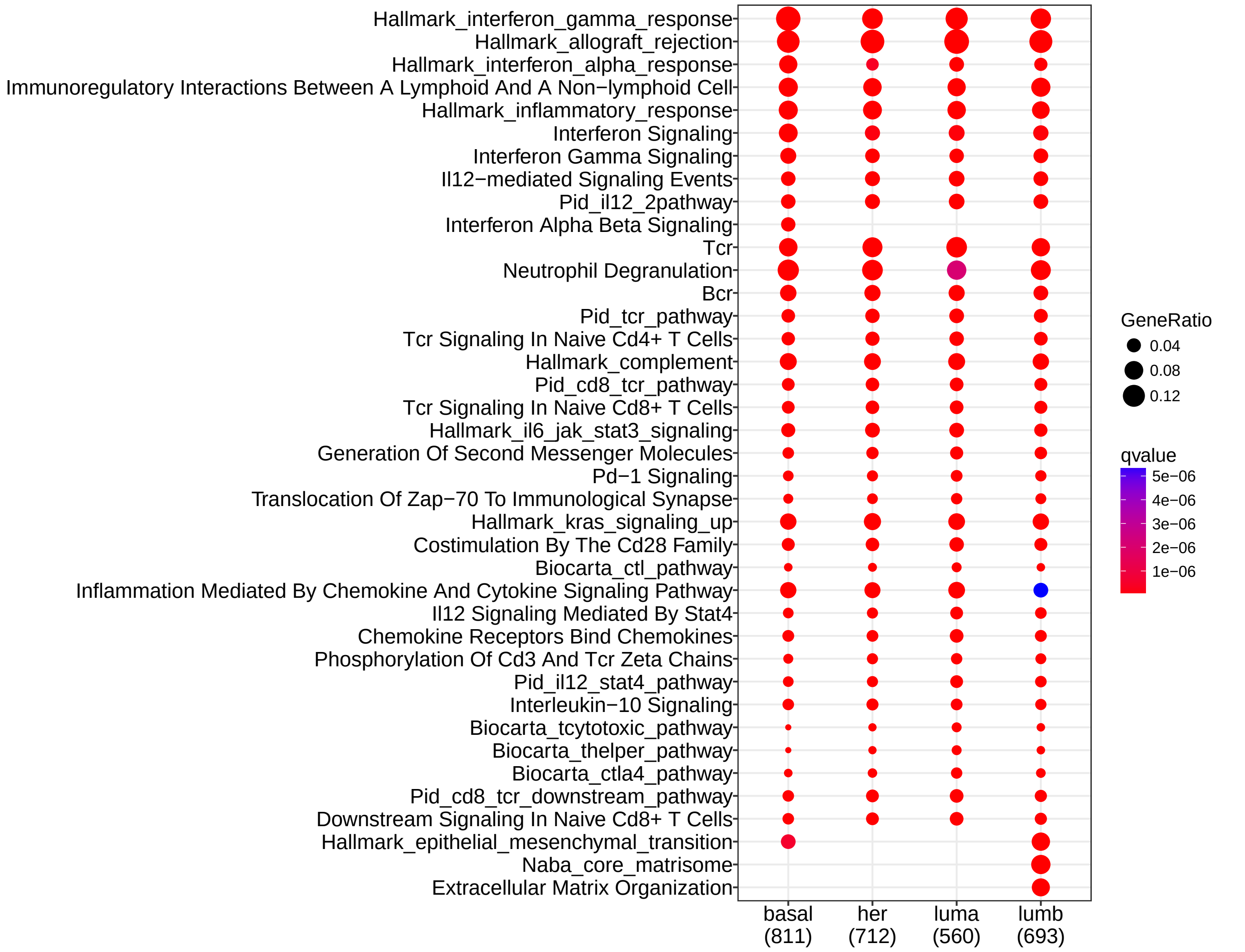

Figure S6: The dot plot for the most significant 27 enriched pathway which were filtered out by p-value cutoff 0.05 and FDR cutoff 1×10^{-4}. Dots in the plot are color coded depending upon the relevant FDR value. Color gradient changes from red(low FDR value, high enrichment) to blue(high FDR value, low enrichment). Dot size depends on the gene ratio, ratio of enriched genes to identified genes in the pathway. Number of identified genes in each subtypes were provided in parenthesis.

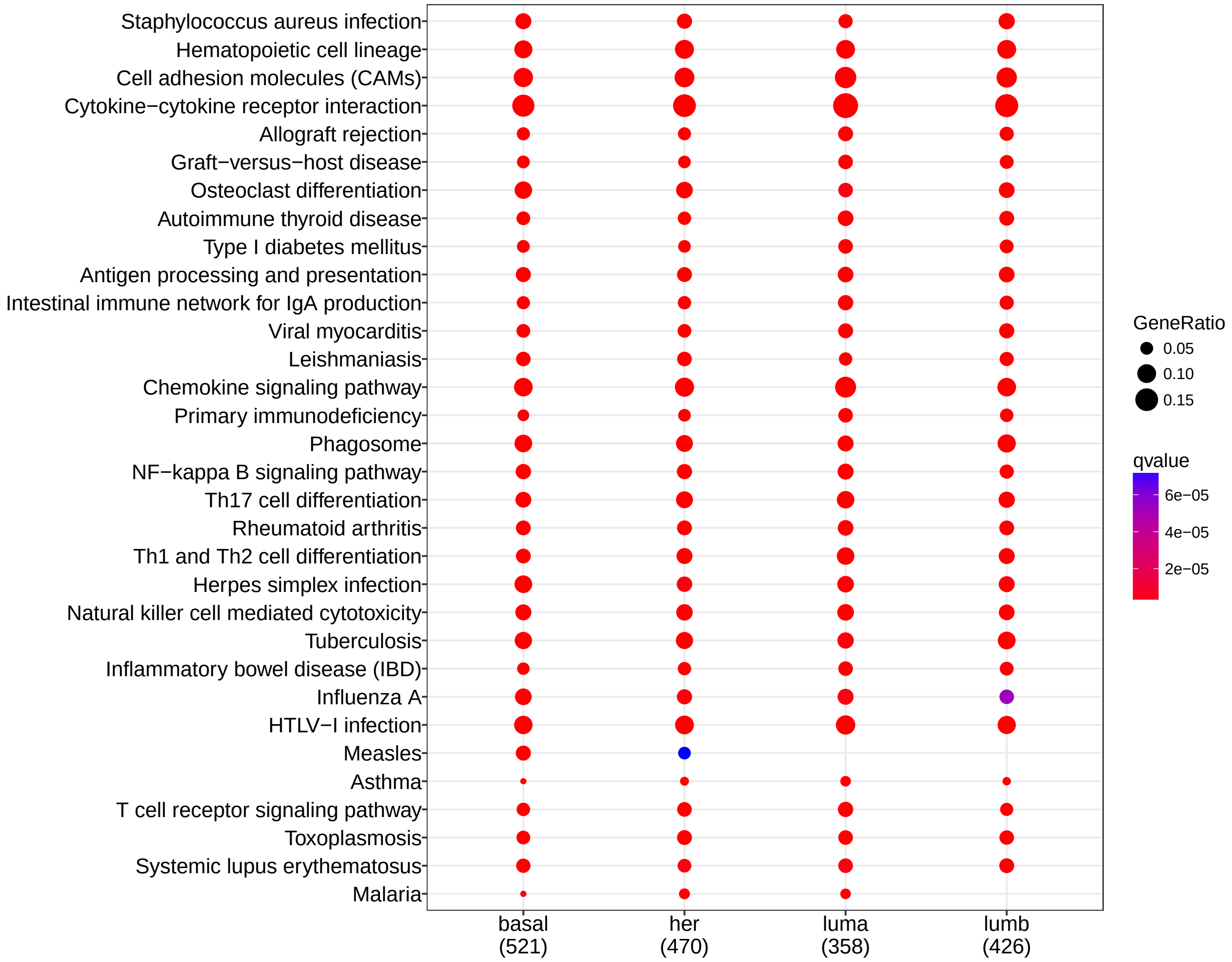

Figure S7: The dot plot for the most significant 27 enriched KEGG pathway which were filtered out by p-value cutoff 0.05 and FDR cutoff 1×10^{-4}. Dots in the plot are color coded depending upon the relevant FDR value. Color gradient changes from red(low FDR value, high enrichment) to blue(high FDR value, low enrichment). Dot size depends on the gene ratio, ratio of enriched genes to identified genes in the pathway. Number of identified genes in each subtypes were provided in parenthesis.

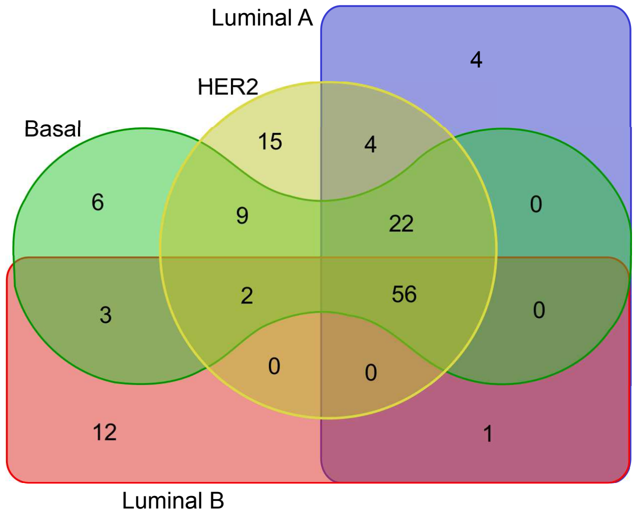

Figure S8: Venn diagram displaying the distribution of enriched pathways (p-value ≤ 0.05 and FDR $\leq 1 \times 10^{-4}$.) over the subtypes.

Table S7: List of subtype specific enriched pathways. Bonferroni corrected p values are provided and all listed pathways are above FDR cutoff 1×10^{-4}.

**Subtypes**	**List of Subtype Specific Pathways**	**p-value**
Luminal A	• Constitutive Signaling By Aberrant Pi3K In Cancer	2.84×10^{-6}
	• Biocarta il17 Pathway	5.22×10^{-6}
	• Pid il2 1 Pathway	5.33×10^{-6}
	• Pid TxA2 Pathway	7.90×10^{-6}
Luminal B	• Naba Core Matrisome	3.58×10^{-20}
	• Extracellular Matrix Organization	2.30×10^{-13}
	• ECM Glycoproteins	2.66×10^{-10}
	• Naba Proteoglycans	2.51×10^{-8}
	• Formation	6.86×10^{-8}
	• Collagen Biosynthesis And Modifying Enzymes	1.19×10^{-7}
	• Integrin Signalling Pathway	5.37×10^{-7}
	• Assembly Of Collagen Fibrils And Other Multimeric Structures	1.89×10^{-6}
	• Pid Integrin1 Pathway	2.99×10^{-6}
	• β1 Integrin Cell Surface Interactions	2.99×10^{-6}
	• Pid αvβ3 Integrin Pathway	3.42×10^{-6}
	• Pid Syndecan 1 Pathway	6.38×10^{-6}
Basal	• Interferon Alpha Beta Signaling	7.20×10^{-23}
	• Antigen Presentation: Folding, Assembly And Peptide Loading Of Class I MHC	1.05×10^{-9}
	• ER-phagosome Pathway	2.52×10^{-8}
	• BCR Signaling Pathway	2.84×10^{-8}
	• Biocarta Complement Pathway	8.14×10^{-8}
	• Pertussis	1.17×10^{-6}
	• Complement Cascade	7.16×10^{-6}
HER2	• Integrin Cell Surface Interactions	3.68×10^{-9}
	• SA MMP Cytokine Connection	2.62×10^{-8}
	• Pid αmβ2 Neutrophils Pathway	2.98×10^{-7}
	• Class I Pi3k Signaling Events	7.47×10^{-7}
	• Il27-Mediated Signaling Events	9.33×10^{-7}
	• Signaling Events Mediated By Stem Cell Factor Receptor (c-kit)	9.86×10^{-7}
	• Calcineurin-Regulated Nfat-Dependent Transcription In Lymphocytes	2.24×10^{-6}
	• CCR1	4.27×10^{-6}
	• Pid GMCSF Pathway	5.01×10^{-6}
	• DAP12 Interactions	7.27×10^{-6}
	• αmβ2 Integrin Signaling	8.24×10^{-6}
	• JAK/STAT Signaling Pathway	9.25×10^{-6}
	• AGE-RAGE Signaling Pathway in Diabetic Complications	1.64×10^{-5}

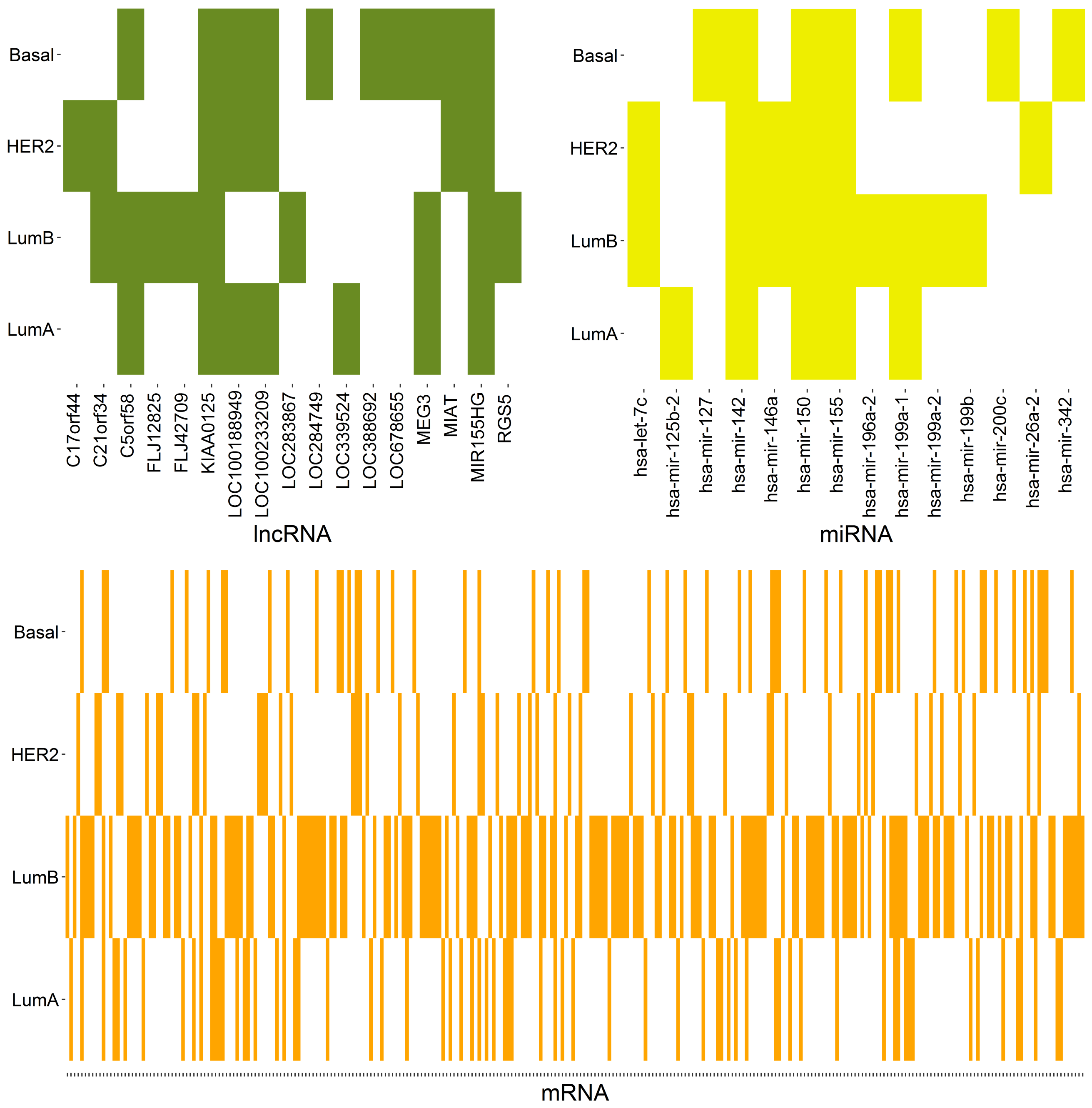

Figure S9: **Distribution of the prognostic RNAs in breast cancer subtypes** Colored cells indicate the RNA is discovered in the given subtype, while white color indicates it is not. To keep the figure simpler and easier to read, list of prognostic mRNAs provided in Supp. File 3.

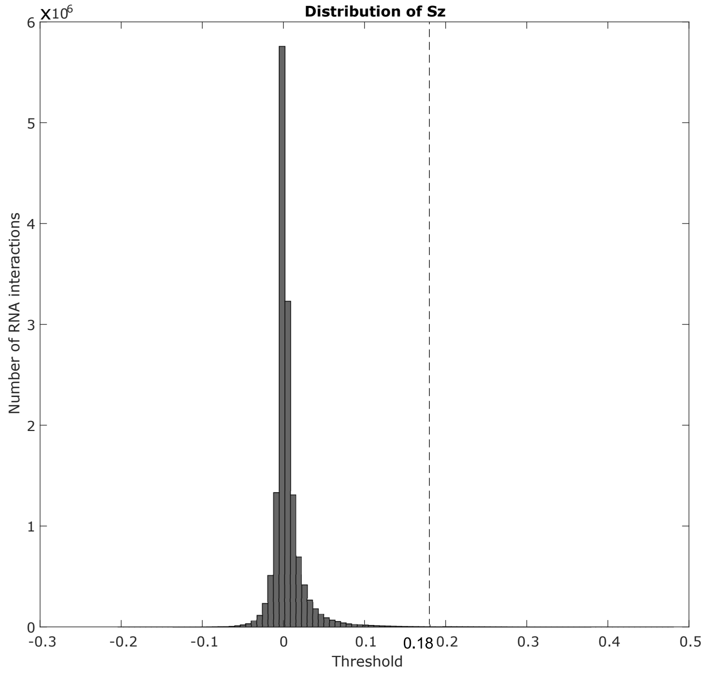

Figure S10: The distribution of the S_z values for all tested RNA triplets. 99th percentile is illustrated with a red dashed line.

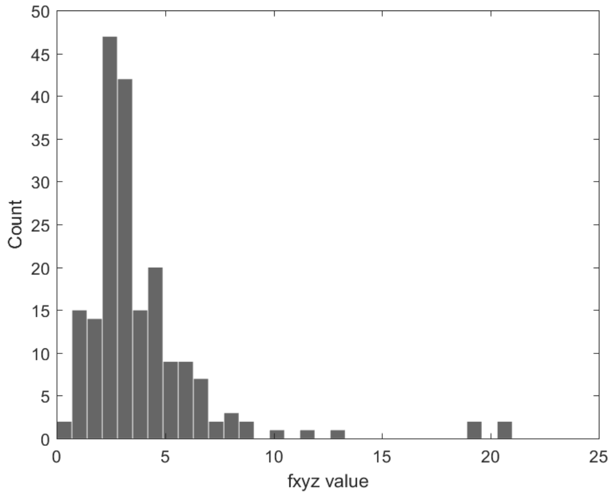

Figure S11: The distribution of the f_{xyz} scores of the prognostic ceRNA interactions.

References

[1] Wang L, Park HJ, Dasari S, Wang S, Kocher JP, Li W. CPAT: Coding-Potential Assessment Tool using an alignment-free logistic regression model. Nucl. Acids Res., 2013; 41(6): e74.

[2] Kong L, Zhang Y, Ye Z, Liu X, Zhao S, Wei L, Gao G. CPC: assess the protein-coding potential of transcripts using sequence features and support vector machines. Nucl. Acids Res. 2007;35, suppl 2 :W345-W349.

[3] Shannon P, Markiel A, Ozier O, Baliga NS, Wang JT, Ramage D, Amin N, Schwikowski B, Ideker T. Cytoscape: a software environment for integrated models of biomolecular interaction networks Genome Research 2003 Nov; 13(11):2498-504
